# Ultrasound features for identifying cancer-associated fibroblasts expression in papillary thyroid carcinoma

**DOI:** 10.3389/fendo.2026.1914939

**Published:** 2026-10-19

**Authors:** Tingting Li, Jiping Xue, Xueting Li, Changjuan Hao, Zhexia Zhao

**Affiliations:** 1 Department of Ultrasound, Third Hospital of Shanxi Medical University, Shanxi Bethune Hospital, Shanxi Academy of Medical Sciences, Tongji Shanxi Hospital, Taiyuan, China; 2 Department of Pathology, Third Hospital of Shanxi Medical University, Shanxi Bethune Hospital, Shanxi Academy of Medical Sciences, Tongji Shanxi Hospital, Taiyuan, China

**Keywords:** cancer-associated fibroblasts, papillary thyroid cancer, diagnostic factor, shear-wave elastography, ultrasonography

## Abstract

**Background:**

Cancer-associated fibroblasts (CAFs) play a critical role in tumor proliferation, invasion, and metastasis, and are considered an important indicator of aggressiveness in papillary thyroid carcinoma (PTC). However, non-invasive assessment of CAFs activation *in vivo* remains challenging. This study aimed to investigate the association between preoperative ultrasound (US) features and the expression levels of CAFs-related markers in PTC, and to assess the utility of US parameters for identifying high CAFs expression.

**Methods:**

This retrospective study included 158 patients with pathologically confirmed PTC who underwent preoperative conventional US and shear wave elastography (SWE). Immunohistochemical staining for fibroblast activation protein (FAP) and α-smooth muscle actin (α-SMA) was performed as markers of CAFs. US parameters were compared between the low and high CAFs expression groups. Correlations between the US parameters and FAP/α-SMA immunoreactivity scores (IRS) were assessed using Spearman’s correlation analysis. Multivariate logistic regression analysis was conducted to identify independent US factors associated with high CAFs expression, and their diagnostic performance was evaluated using receiver operating characteristic (ROC) curves.

**Results:**

Significant differences were observed in tumor size, suspected extrathyroidal extension, Emax, Emean, and ESD between low and high expression groups of FAP and α-SMA (all *P* < 0.001). Blood flow grade also differed significantly between FAP expression groups (*P* < 0.001). The IRS of both FAP and α-SMA correlated most strongly with Emax (r = 0.555 and 0.575, respectively; both *P* < 0.001).

Multivariate analysis identified blood flow grade 2–3 (OR = 2.513, 95% CI: 1.049–6.025, *P* = 0.039) and Emax (OR = 1.038, 95% CI: 1.003–1.074, *P* = 0.033) were independently associated with high FAP expression, whereas only Emax (OR = 1.048, 95% CI: 1.012–1.085, *P* = 0.009) was independently associated with high α-SMA expression. Emax showed good diagnostic performance for both FAP (AUC = 0.799) and α-SMA (AUC = 0.819). Adding blood flow grade to Emax did not significantly improve AUC for identifying high FAP expression (0.826 vs. 0.799, *P* = 0.564).

**Conclusion:**

US parameters, particularly Emax, are valuable for non-invasively assessing CAFs activation in PTC, which could help to guide individualized preoperative clinical decision-making.

## 1 Introduction

Thyroid cancer is the most common malignant tumor of the endocrine system, and its global incidence has risen rapidly in recent years (1). Papillary thyroid carcinoma (PTC) is the most common pathological type of thyroid cancer. Although the overall prognosis of PTC is relatively favorable, some tumors still exhibit highly aggressive behavior such as extrathyroidal invasion and lymph node (LN) metastasis (2, 3), leading to clinical dilemmas of under-treatment or over-treatment. Therefore, how to accurately assess the biological behavior of PTC and optimize treatment strategies is still a key issue needing resolution in clinical practice.

The occurrence and development of tumors depend not only on the cancer cells themselves but also on their surrounding microenvironment. Cancer-Associated Fibroblasts (CAFs), an important component of the tumor microenvironment (TME), have been proven to play a significant role in tumor proliferation, invasion, and metastasis (4, 5). CAFs can serve as an important indicator for evaluating the aggressive biological behavior of PTC, and their high expression is considered a risk factor for extrathyroidal extension, LN metastasis, and multifocality in PTC (6–8). Hypothetically, if high CAFs activity are non-invasively identifiable before surgery, such information might prompt clinicians to exercise heightened vigilance during intraoperative inspection of aggressive tumor behavior and could conceivably guide more tailored decisions regarding the extent of resection. Furthermore, CAFs may become new therapeutic targets for thyroid cancer (9, 10). Therefore, assessing the activation level of CAFs in thyroid cancer has important clinical significance.

The activation status of CAFs can be reflected by the expression levels of relevant immunohistochemical markers (11, 12). In PTC, fibroblast activation protein (FAP) and α-smooth muscle actin (α-SMA) are recognized as important biomarkers for CAFs (13, 14). At present, the detection of CAFs-related biomarkers is complicated and costly. Moreover, evaluation of CAFs can only be performed using postoperative tissue specimens and cannot be achieved *in vivo*, leading to clinical lag.

Ultrasound (US) is the first-line imaging modality for the diagnosis and preoperative evaluation of thyroid cancer. Although US features are closely correlated with pathological alterations in thyroid carcinoma, few studies have investigated the association between US features and the activation level of CAFs in thyroid cancer. Shear wave elastography (SWE) provides quantitative measurements of tissue stiffness, which may complement conventional US findings. Accordingly, this study aimed to investigate the relationship between conventional and elastography US features of PTC and the expression levels of FAP/α-SMA, and to identify independent factors associated with high CAFs expression, thereby providing a new perspective for the preoperative assessment of PTC biological behavior.

## 2 Materials and methods

### 2.1 Patients

Consecutive patients who underwent thyroid surgery and were pathologically diagnosed with PTC in our hospital from December 2023 to December 2024 were retrospectively analyzed. All patients underwent thyroid conventional US and SWE before surgery (less than 1 month from the time of surgery). Exclusion criteria: 1) incomplete clinical data; 2) unclear pathological immunohistochemical staining; 3) incomplete US images data; 4) thyroid therapy before surgery including radiofrequency ablation, radiotherapy or chemotherapy. The patient study flowchart is shown in **Figure 1**. This study was approved by the Ethics Committee of our hospital. Informed consent was waived due to the retrospective study.

**Figure 1:**
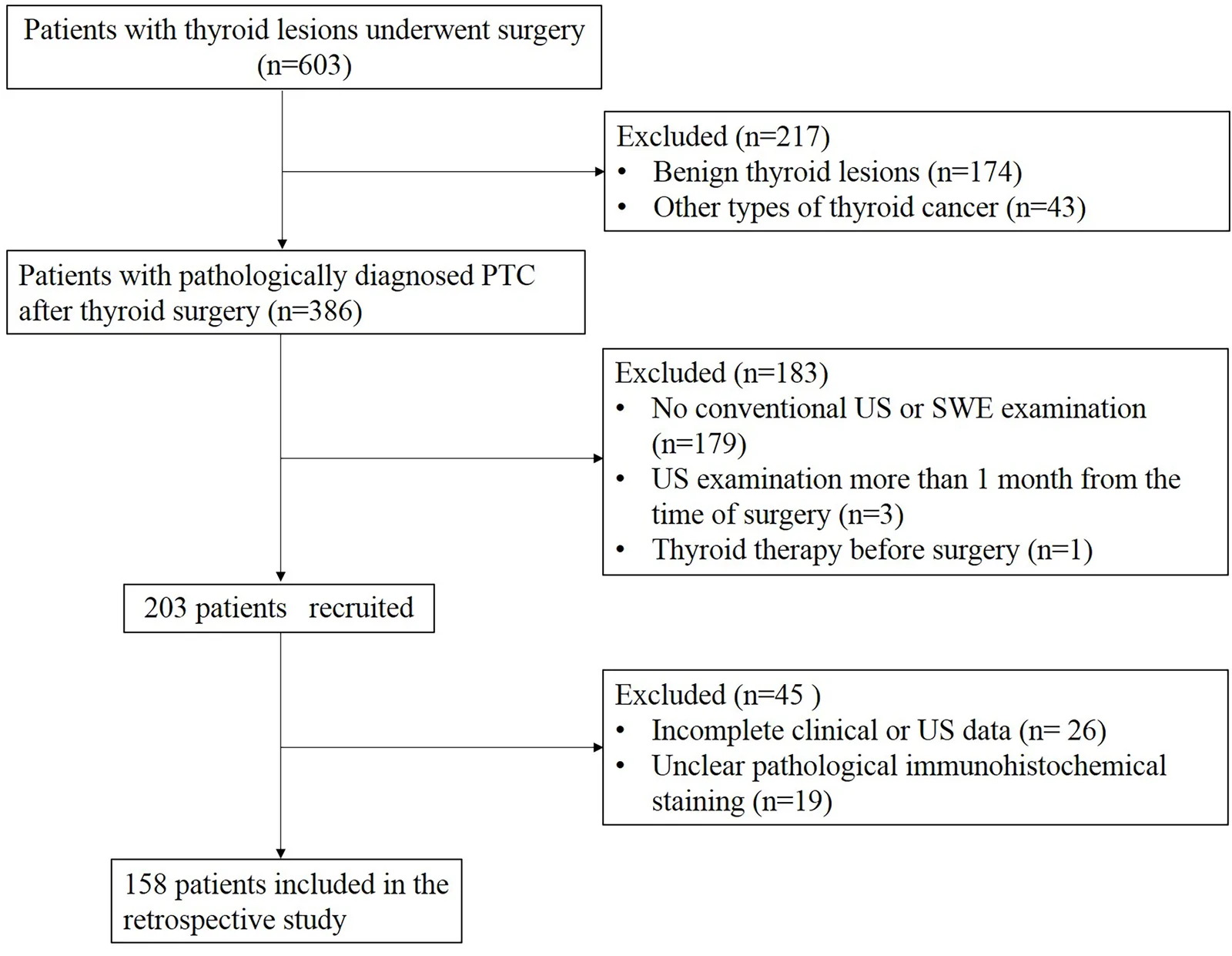
Flowchart of patient selection.

### 2.2 US image acquisition and analysis

Each patient underwent conventional US and SWE using the Aixplorer ultrasound system (SuperSonic Imagine, Aix-en-Provence, France) with a linear probe (frequency range 4–15 MHZ). All US examination was conducted by the same sonographer with more than 10 years of experience in thyroid US diagnosis who had trained in elastography. Conventional US features of PTC were evaluated, including tumor maximum size, echogenicity (isoechoic or hypoechoic, marked hypoechoic), margins (circumscribed, uncircumscribed), aspect ratio (wider-than-taller ≤ 1, taller than-wider > 1), punctate echogenic foci (absence, presence); suspected extrathyroidal extension (absent, present or suspicious).

Tumor blood supply was graded according to Adler flow grade classification: 0 (no flow), 1 (poor flow), 2 (medium flow), and grade 3 (rich flow) (15). After conventional US features were recorded, the SWE mode was switched. The region of interest was set to include the thyroid lesion and surrounding tissue. During the SWE image acquisition, the scan pressure applied by the operator was as small as possible, and the probe was kept fixed to ensure stable image quality. The circular elastic sampling frame was adjusted to cover the entire lesion area. The US system automatically calculated the maximum (Emax), mean (Emean), minimum (Emin), and standard deviation (ESD) of the elasticity values. At least three images were acquired for each lesion, and the elasticity values were averaged. All US parameters were independently evaluated by two experienced sonographers who were blinded to the pathological results. Any disagreements were resolved by consensus with a third sonographer.

### 2.3 Immunohistochemical staining and scoring of CAFs

The samples were fixed with 10% neutral formalin, embedded in paraffin and sectioned (with a thickness of 4 μm). Immunohistochemical staining was performed using antibodies against α-SMA (clone No. ZM-0003, Zhongshan Golden Bridge Biotechnology, China) and FAP (clone No. ab207178, Abcam, UK) according to the instructions. Scoring was adopted to immunoreactivity score (IRS). Staining intensity was scored as 0 (negative), 1 (weak), 2 (moderate), or 3 (strong). The proportion of positive cells was scored as 0 (<5%), 1 (5%–25%), 2 (25%–50%), 3 (50%–75%), or 4 (>75%). CAFs expression was specifically evaluated in the stromal compartment. The final IRS was calculated by multiplying the intensity and proportion scores. For each section, five representative fields were captured at ×200 magnification by an experienced pathologist blinded to the clinical and US data. The same senior pathologist re-evaluated a random subset of 40 cases one month after the initial reading, under blinded conditions. Cases were divided into high- and low-expression groups using an IRS cutoff of ≥ 6, based on the median IRS value (8).

### 2.4 Clinical and pathological data collection

Clinical and pathological data, including gender, age, cervical lymph node (LN) metastasis, extrathyroidal extension, T classification (based on the 8th edition of the AJCC staging system), and the presence or absence of coexisting Hashimoto’s thyroiditis, were collected from medical records.

### 2.5 Statistical analysis

Statistical analysis was performed using SPSS software (version 25.0, IBM Corporation, Armonk, NY, USA). Categorical variables were expressed as percentages and compared using the Chi-square test. Continuous variables were expressed as mean ± standard deviation or median (interquartile range) and compared using the independent t-test or Mann-Whitney U test, as appropriate. The correlation between CAFs immunohistochemical score and US parameters was analyzed using Spearman’s rank correlation analysis. Multivariate logistic regression analysis was used to identify independent risk factors for high CAFs expression. Receiver operating characteristic (ROC) curves were constructed, and the area under the curve (AUC) was calculated. The AUCs of different US parameters were compared using the Z-test. Intra-observer agreement was evaluated by the intra-class correlation coefficient (ICC). *P* < 0.05 was considered statistically significant.

## 3 Results

### 3.1 Clinical and pathological characteristics

The clinical and pathological characteristics of all patients are summarized in **Table 1**. Of these patients, there were 117 females (74.1%) and 41males (25.9%), with a mean age of 45.84 ± 11.81 years. Based on postoperative pathology, 68 patients (43.0%) were diagnosed with extrathyroidal extension, 78 patients (49.4%) were diagnosed with cervical LN metastasis, and 101 patients (63.9%) were T3–T4. Overall, 56(35.4%) patients co-existed with Hashimoto’s thyroiditis.

**Table 1:** Clinical and pathological characteristics of all patients.

Clinical and pathological characteristics	No.
Sex, n (%)	
male	41 (25.9)
female	117 (74.1)
Age (years)	45.84 ± 11.81
Extrathyroidal extension, n (%)	
yes	68 (43.0)
no	90 (57.0)
Cervical LN metastasis, n (%)	
yes	78 (49.4)
no	80 (50.6)
T classification, n (%)	
1-2	57 (36.1)
3-4	101(63.9)
Hashimoto’s thyroiditis, n (%)	
absence	102 (64.6)
presence	56 (35.4)

### 3.2 Relationship between US parameters and FAP/α-SMA expression levels

Immunohistochemical staining showed that both FAP and α-SMA were mainly expressed in the stroma of PTC, presenting as brownish-yellow staining. Intra-observer reproducibility in the immunohistochemical evaluation was excellent, with ICC of 0.934 (95% CI: 0.879–0.964) for FAP and 0.939 (95% CI: 0.888–0.967) for α-SMA.

The corresponding histopathological and US images are shown in **Figure 2**. Significant differences were observed in tumor maximum size, suspected extrathyroidal extension, blood flow grade, Emax, Emean and ESD between the low and high expression groups of FAP (all *P* < 0.001). Significant differences were also observed in tumor maximum size, suspected extrathyroidal extension, Emax, Emean, and ESD between the low and high expression groups of α-SMA (all *P* < 0.001). However, no significant differences were found in echogenicity, margins, aspect ratio, punctate echogenic foci, or Emin (all *P* > 0.05; **Table 2**).

**Figure 2:**
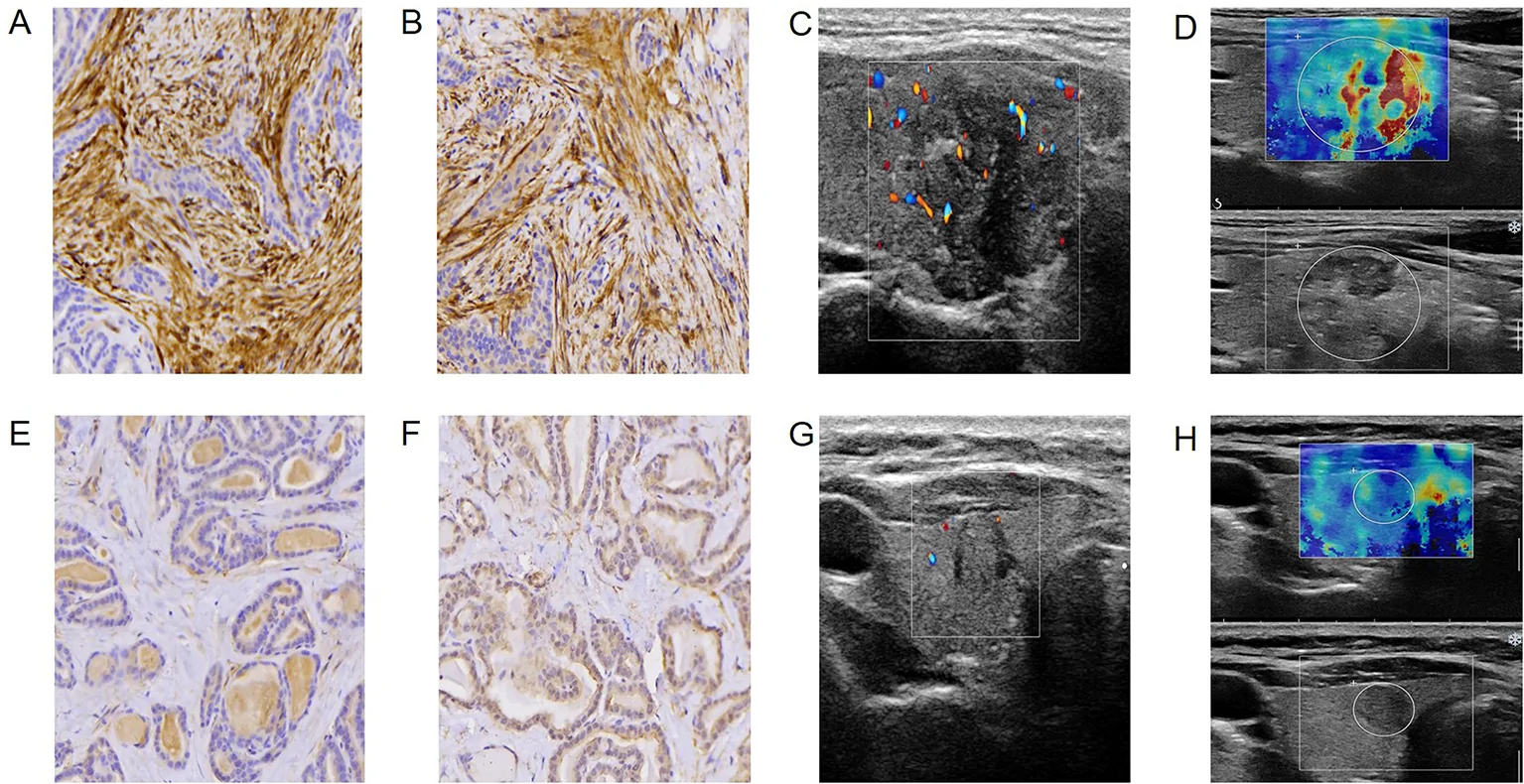
Histopathological and ultrasound images of PTC lesions with different CAFs expression. **(A, B)** FAP and α-SMA immunostaining from left to right (scores: 3 × 4 and 3 × 4, respectively; ×200) in a 2.5 × 2.0 cm tumor. **(C)** Conventional ultrasound of the same lesion. **(D)** SWE image of the same lesion (Emax = 148.9 kPa). **(E, F)** FAP and α-SMA immunostaining from left to right (scores: 1 × 1 and 1 × 1, respectively; ×200) in a 0.8 × 0.7 cm tumor. **(G)** Conventional ultrasound of the same lesion. **(H)** SWE image of the same lesion (Emax = 46.9 kPa).

**Table 2:** Comparison of ultrasound parameters between FAP/α-SMA low- and high-expression groups.

Ultrasound parameters	FAP		*P*-value	α-SMA		*P*-value
	Low expression (n=75)	High expression (n=83)		Low expression (n=79)	High expression (n=79)	
Maximum size (cm)	0.83 ± 0.45	1.25 ± 0.66	<0.001	0.84 ± 0.41	1.26 ± 0.69	<0.001
Echogenicity, n (%)			0.199			0.217
isoechoic or hypoechoic	25 (33.3)	20 (24.1)		19 (24.1)	26 (32.9)	
marked hypoechoic	50 (66.7)	63 (75.9)		60 (75.9)	53 (67.1)	
Margins, n (%)			0.633			0.175
circumscribed	8 (10.7)	7 (8.4)		10 (12.7)	5 (6.3)	
uncircumscribed	67 (89.3)	76 (91.6)		69 (87.3)	74 (93.7)	
Aspect ratio, n (%)			0.072			0.101
wider-than-taller ≤ 1	23 (30.7)	37 (44.6)		25 (31.6)	35 (44.3)	
taller than-wider > 1	52 (69.3)	46 (55.4)		54 (68.4)	44 (55.7)	
Punctate echogenic foci, n (%)			0.220			0.147
absence	47 (62.7)	44 (53.0)		50 (63.3)	41 (51.9)	
presence	28 (37.3)	39 (47.0)		29 (36.7)	38 (48.1)	
Suspected extrathyroidal extension, n (%)			<0.001			<0.001
absence	65 (86.7)	45 (54.2)		66 (83.5)	43 (55.1)	
presence/suspicious	10 (13.3)	38 (45.8)		13 (16.5)	35 (44.9)	
Blood flow grade, n (%)			<0.001			0.176
0-1	61 (81.3)	45 (54.2)		57 (72.2)	49 (62.0)	
2-3	14 (18.7)	38 (45.8)		22 (27.8)	30 (38.0)	
Emax (kpa)	50.60 ± 22.62	97.01 ± 55.45	<0.001	50.90 ± 25.87	99.05 ± 54.42	<0.001
Emean (kpa)	27.33 ± 13.62	39.84 ± 21.74	<0.001	27.48 ± 13.87	40.24 ± 21.87	<0.001
Emin (kpa)	12.82 ± 11.10	15.13 ± 16.42	0.307	13.84 ± 11.57	14.22 ± 16.41	0.870
Esd (kpa)	7.90 ± 4.13	15.93 ± 11.11	<0.001	8.01 ± 6.17	16.22 ± 10.32	<0.001

The correlations between CAFs IRS and the significant US parameters are presented in **Table 3**. CAFs IRS was positively correlated with these parameters. Among these, the IRS of both FAP and α-SMA showed the strongest correlation with Emax (Spearman’s r = 0.555 and 0.575, respectively, both *P* < 0.001).

**Table 3:** Correlation between ultrasound parameters and CAFs immunoreactivity scores.

Ultrasound parameters	FAP		α-SMA	
	Spearman’s r	*P*-value	Spearman’s r	*P*-value
Maximum size	0.382	<0.001	0.371	<0.001
Suspected extrathyroidal extension	0.310	<0.001	0.295	<0.001
Blood flow grade	0.338	<0.001	–	–
Emax	0.555	<0.001	0.575	<0.001
Emean	0.378	<0.001	0.365	<0.001
Esd	0.475	<0.001	0.516	<0.001

### 3.3 Multivariate logistic regression analysis of US parameters for identifying high FAP/α-SMA expression

Multivariate analysis revealed that blood flow grade 2–3 (OR = 2.513, 95% CI: 1.049–6.025, *P* = 0.039) and Emax (OR = 1.038, 95% CI: 1.003–1.074, *P* = 0.033) were independently associated with high FAP expression, whereas only Emax (OR = 1.048, 95% CI: 1.012–1.085, *P* = 0.009) remained independently associated with high α-SMA expression (**Table 4**).

**Table 4:** Multivariate analysis of ultrasound parameters for high CAFs expression in PTC.

Variable	FAP			α-SMA		
	OR	95% CI	*P*-value	OR	95% CI	*P*-value
Maximum size	0.711	0.271-1.865	0.488	0.828	0.336-2.041	0.681
Suspected extrathyroidal extension	2.261	0.864-5.917	0.094	1.517	0.595-3.866	0.382
Blood flow grade	2.513	1.049-6.025	0.039	–	–	–
Emax	1.038	1.003-1.074	0.033	1.048	1.012-1.085	0.009
Emean	1.010	0.979-1.042	0.537	1.002	0.972-1.033	0.898
Esd	0.973	0.845-1.119	0.072	0.955	0.831-1.098	0.520

### 3.4 Discriminative ability of US parameters for high FAP/α-SMA expression

ROC analysis demonstrated that Emax had good discriminative ability for both high FAP expression (AUC = 0.799, 95% CI: 0.731–0.866) and high α-SMA expression (AUC = 0.819, 95% CI: 0.750–0.876), with no statistically significant difference between the two AUCs (*Z* = 0.420, *P* = 0.674). Blood flow grade showed lower diagnostic performance than Emax for high FAP expression (AUC = 0.636, 95% CI: 0.549–0.722, *Z* = 3.622, *P* < 0.001). The combination of Emax and blood flow grade improved the diagnostic performance for high FAP expression (AUC = 0.826, 95% CI: 0.764–0.888); however, this improvement was not statistically significant compared with Emax alone (*Z* = 0.576, *P* = 0.564) (**Figure 3**).

**Figure 3:**
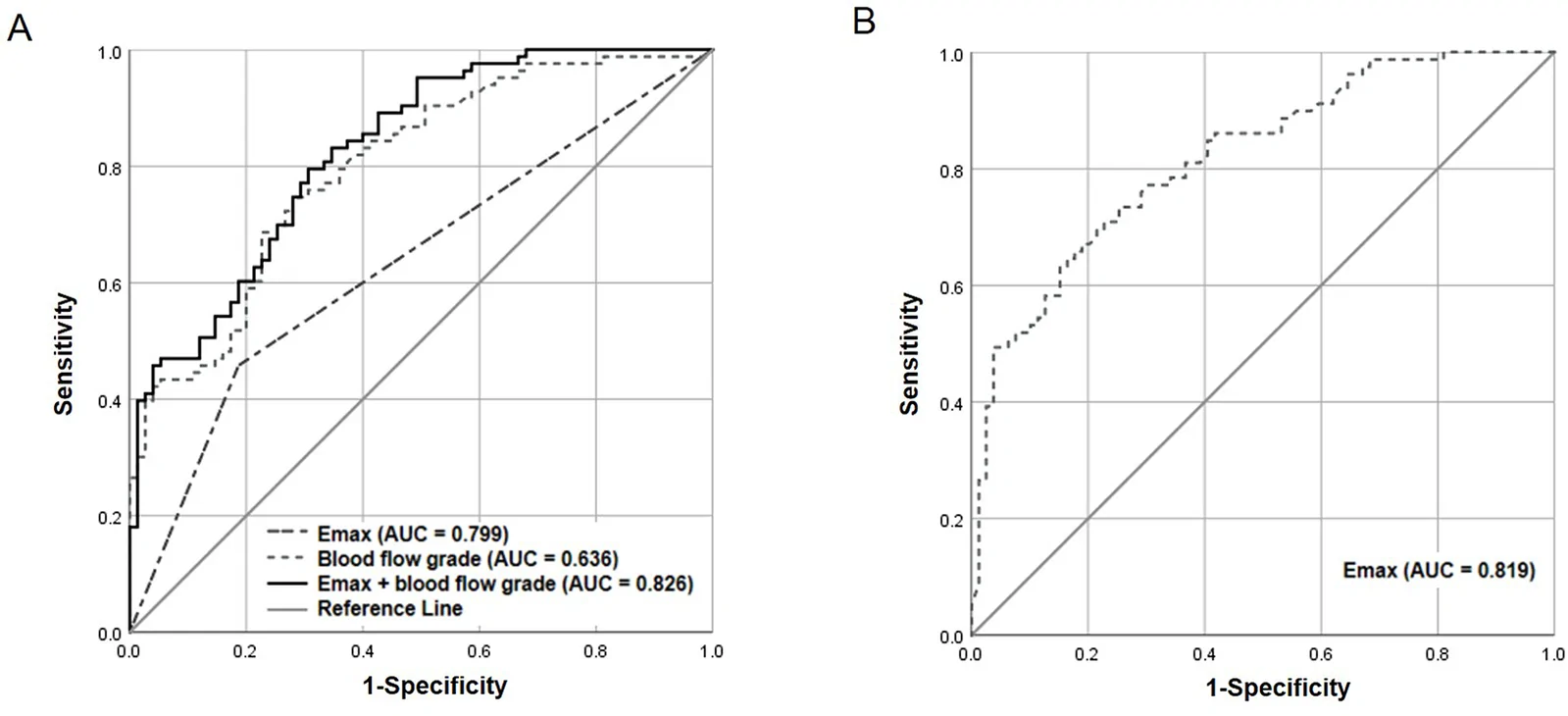
Receiver operating characteristic (ROC) curves for identifying high expression of FAP and α-SMA. **(A)** ROC curves for identifying high FAP expression using Emax, blood flow grade, and their combination. **(B)** ROC curves for identifying high α-SMA expression using Emax.

## 4 Discussion

CAFs are core stromal components of the TME and play a critical role in regulating tumor proliferation, invasion, and progression of PTC. However, traditional assessment of CAFs activation rely on postoperative immunohistochemical staining, which is invasive and fail to enable *in vivo* real-time evaluation, limiting its clinical applicability. Therefore, this study focused on the relationship between CAFs expression levels and US features, and further identified independent US factors associated with high CAFs activation, which may provide a practical non-invasive method for clinically estimating CAFs activation.

Among the evaluated US parameters, Emax showed the strongest correlation with both IRS of FAP and α-SMA. Multivariate analysis identified Emax as an independent risk factor for high FAP/α-SMA expression, indicating that increased tumor stiffness is associated with higher CAFs activation level in PTC. SWE can provide real-time, non-invasive information on tissue stiffness. Previous studies have shown that SWE parameters can distinguish between benign and malignant thyroid nodules and predict the aggressiveness of PTC (16, 17). However, these studies fail to elucidate the mechanistic basis linking elastography parameters to tumor biological behaviors. Previous research has shown that increased stiffness in thyroid cancer is associated with increased collagen content in the extracellular matrix (ECM) (18, 19). A characteristic feature of CAFs is their markedly enhanced capacity to produce and remodel the ECM. Activated fibroblasts promote stromal fibrosis by secreting ECM components such as collagen, and facilitate ECM remodeling by releasing matrix-modifying enzymes, which alters the composition and cross-linking status of the tumor stroma to increased tumor stiffness (20, 21). Based on this, we hypothesize that increased tissue stiffness resulting from CAFs activation may represent a potential pathological basis for the association between elastography parameters and CAFs expression in PTC. It should be emphasized, however, that our study is observational and therefore cannot establish causality; this proposed CAF–ECM–stiffness link remains a hypothesis. Further mechanistic investigations are required to validate this relationship.

Previous studies have shown that CAFs exist as distinct subtypes, exhibiting diversity in origin, phenotype, and function (22, 23). This study found that higher blood flow grade was independently associated with high FAP expression but was not significantly associated with α-SMA expression. We speculate that tumor angiogenesis in PTC may be more closely associated with FAP-positive CAFs than with α-SMA-positive CAFs, although this remains to be validated. This further confirms the heterogeneity of CAFs marked by FAP and α-SMA in the remodeling of the PTC microenvironment.

Interestingly, none of the conventional grayscale US features were retained in the multivariate regression model. This finding suggests that grayscale US, although useful for describing macroscopic tumor morphology such as aspect ratio, margins, and echogenicity, has limited capacity in assessing the TME.

ROC analysis further demonstrated that Emax exhibited good diagnostic performance for high FAP and α-SMA expression, with AUC values of 0.799 and 0.819, respectively. Incorporating blood flow grade into Emax slightly increased the AUC for identifying high FAP expression to 0.826; however, this improvement was not statistically significant compared to using Emax alone. These results indicate that Emax may be used as a simple and reliable indicator for preoperative assessment of CAFs activation in PTC.

Our study focused on CAFs expression in PTC and did not include clinical laboratory parameters such as circulating fibroblast growth factor 21 (FGF21). However, recent evidence indicates that serum FGF21 levels correlate with PTC aggressiveness and its sonographic findings (24, 25). Therefore, combining serum FGF21 levels, US, and CAFs biomarkers may enable more comprehensive risk stratification for PTC in future investigations.

## 5 Conclusion

US parameters, including elasticity values and blood flow grade, can effectively reflect CAFs expression in PTC, among which Emax demonstrated the greatest potential as a non-invasive biomarker for CAFs activation, which may be used as supplementary preoperative risk information.

This study had several limitations. First, selection bias may be present, and an *a priori* sample size calculation was not performed, owing to the single-center retrospective design. Subgroup analysis exploring the impact of HT on this relationship could not be conducted due to the limited sample size. Larger cohorts are warranted for further validation in future investigations. Second, Doppler grading was evaluated subjectively, which may introduce inter-observer variability. Third, clinical outcome data were not available in this retrospective cohort; future studies with complete outcome data and external validation cohorts are required to corroborate our findings. Fourth, we did not include other imaging modalities such as contrast-enhanced ultrasound and superb microvascular imaging, which may offer complementary information on CAFs activation. Fifth, the use of only FAP and α-SMA as CAFs markers may not fully capture CAFs heterogeneity. Future animal and cellular studies are needed to further clarify the link between CAFs and the US features.

## Data Availability

The original contributions presented in the study are included in the article/supplementary material. Further inquiries can be directed to the corresponding author.
